# Post-transcriptional regulation of the transcriptional apparatus in neuronal development

**DOI:** 10.3389/fnmol.2024.1483901

**Published:** 2024-12-23

**Authors:** Mohammad Nazim

**Affiliations:** Department of Microbiology, Immunology, and Molecular Genetics, University of California, Los Angeles, Los Angeles, CA, United States

**Keywords:** post-transcriptional regulation, alternative splicing, RNA-binding protein, neuronal development, transcription, epigenetic regulation, transcription factor, histone-modifying enzyme

## Abstract

Post-transcriptional mechanisms, such as alternative splicing and polyadenylation, are recognized as critical regulatory processes that increase transcriptomic and proteomic diversity. The advent of next-generation sequencing and whole-genome analyses has revealed that numerous transcription and epigenetic regulators, including transcription factors and histone-modifying enzymes, undergo alternative splicing, most notably in the nervous system. Given the complexity of regulatory processes in the brain, it is conceivable that many of these splice variants control different aspects of neuronal development. Mutations or dysregulation of splicing and transcription regulatory proteins are frequently linked to various neurodevelopmental disorders, highlighting the importance of understanding the role of neuron-specific alternative splicing in maintaining proper transcriptional regulation in the brain. This review consolidates current insights into the role of alternative splicing in influencing transcriptional and chromatin regulatory programs in neuronal development.

## Introduction

Alternative splicing (AS) plays a major role in expanding proteomic diversity by allowing a limited number of eukaryotic genes to generate multiple protein variants, and thereby significantly enhancing the functional complexity of the genome. Current knowledge indicates that roughly 95% of the pre-mRNA transcripts of human multiexon genes undergo AS (Pan et al., 2008; Wang et al., 2008). AS ensures the appropriate removal of introns and the inclusion or skipping of specific exons through the selective use of splice sites in pre-mRNA transcripts. This process often occurs in a tissue-specific or developmental-stage-specific manner, orchestrated by the binding of specific *trans*-acting splicing regulatory proteins to their cognate *cis*-regulatory elements dispersed in the alternatively spliced exons and/or their flanking introns (Black, 2003; Nazim et al., 2016, 2018; Ohno et al., 2017; Vuong C. K. et al., 2016). As in many other tissues, AS is common for genes involved in the development of the nervous system, where alternatively spliced protein isoforms determine the cell fate decisions and properties of different cell types within the neuronal lineage. Changes in the expression of specific splicing regulatory proteins during neuronal development induce alterations in splicing of a large set of exons (Boutz et al., 2007; Gueroussov et al., 2015; Li et al., 2014; Vuong J. K. et al., 2016). The resulting alternative protein isoforms regulate diverse functions of neuronal development, including transcription, chromatin remodeling, apoptosis, synaptogenesis, and axonogenesis (Lin et al., 2020; Linares et al., 2015; Nazim et al., 2024; Zhang M. et al., 2019; Zheng et al., 2012). The critical role of specific splicing decisions and splicing regulatory factors in the nervous system development and function is becoming increasingly evident. Below, we discuss the molecular mechanisms that govern post-transcriptional regulation of transcriptional and chromatin regulators during neuronal development and highlight several cellular processes where splicing regulation plays a critical role.

## Neuronal alternative splicing programs

Alternative splicing is highly prevalent in complex organisms such as vertebrates, where the brain displays a considerably greater number of alternative splicing events than other tissues (Pan et al., 2008; Xu, 2002; Yeo et al., 2004). Brain-specific alternative splicing programs are highly conserved throughout vertebrate evolution, indicating the functional importance of the alternatively spliced variants (Barbosa-Morais et al., 2012; Merkin et al., 2012). Notably, multiple studies from several groups have highlighted the neocortex as a major site for alternative splicing and demonstrated its effect on cortical development (Belgard et al., 2011; McKee et al., 2005; Zhang et al., 2016, 2014).

The primary machinery for splicing, the spliceosome, determines which pre-mRNA segments will be included or excluded in the mature mRNA. The spliceosome is a dynamic macromolecular RNA-protein complex composed of five RNA subunits (U1, U2, U4, U5, and U6), associated small ribonucleoproteins (RNPs), and a large number of auxiliary factors that assist the spliceosome to recognize splice sites (Black, 2003; Matera and Wang, 2014; Nazim et al., 2018; Vuong C. K. et al., 2016; Wahl et al., 2009; Wilkinson et al., 2020). The spliceosomal assembly process begins when U1 snRNP binds to the 5′ splice site (SS), SF1 protein binds to the branch point (BP), and U2 auxiliary factor heterodimer (U2AF65 and U2AF35) binds to the polypyrimidine tract and the 3′ splice site, respectively. This initial complex formation, known as the E-complex, is ATP-independent. In the next step, SF1 is replaced by U2 snRNP at the BP in an ATP-dependent manner, forming the A-complex. Subsequent recruitment of the U4/U6.U5 tri-snRNPs leads to the formation of the B-complex. At this stage, the spliceosome undergoes extensive remodeling and conformational changes, releasing U1 and U4 snRNPs to form the catalytically active C-complex. Subsequently, the intron forms a lariat structure and is excised, followed by the ligation of the two neighboring exons to complete the splicing reaction.

**Figure 1:**
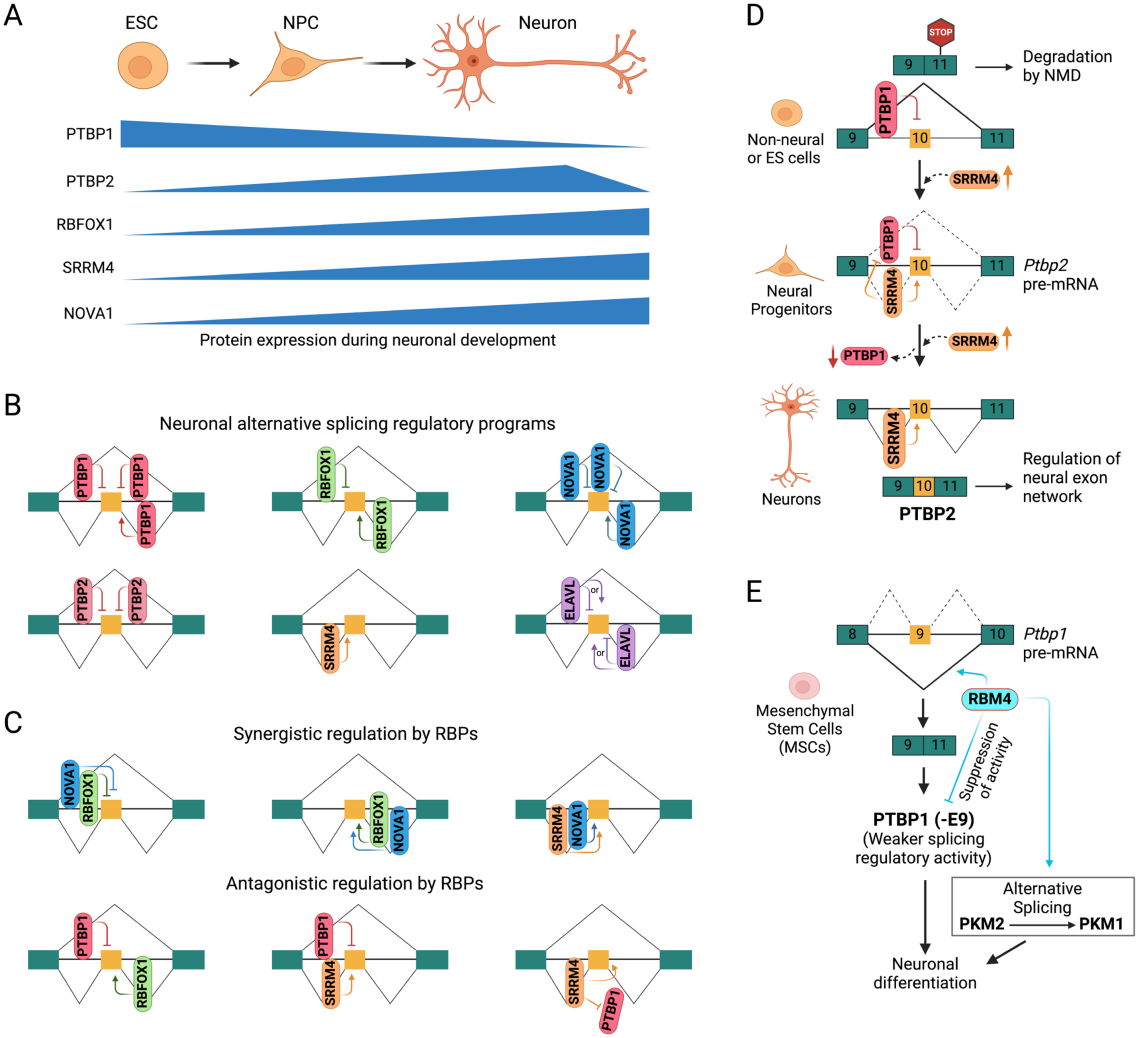
Neuronal splicing regulatory programs. **(A)** Schematic showing developmental stage-specific expression of splicing regulatory RNA binding proteins in embryonic stem cells (ESCs), neural progenitor cells (NPCs), and mature neurons. **(B)** Position/context-dependent alternative splicing regulation by neuronal RNA-binding proteins. Constitutive and alternatively spliced exons are shown as green and orange boxes, respectively. **(C)** Coordinated regulation (synergistic and antagonistic) of alternative splicing by multiple RNA binding proteins. **(D)** Functional antagonism between PTBP1 and SRRM4 in regulating the alternative splicing of PTBP2 exon 10 during neuronal development. **(E)** Functional antagonism between PTBP1 and RBM4 in alternative splicing regulation during neuronal differentiation.

Although most spliceosome components discussed above are ubiquitously expressed, many alternative splicing events are regulated in a developmental-stage-specific or tissue-specific manner. This is achieved by tissue-specific expression of specific splicing regulatory proteins that direct the spliceosome to particular splice sites. Neuron-specific splicing, for example, is controlled by various brain-specific splicing regulatory programs (Figures 1A,B and Table 1). The splicing regulation by neuronal splicing factors is often context-dependent, and multiple RNA-binding proteins can regulate splicing events synergistically or antagonistically (Figure 1C). Recent reviews have extensively discussed the mechanisms and roles of these splicing regulators in brain development (Lara-Pezzi et al., 2017; Lee et al., 2023; Porter et al., 2018; Raj and Blencowe, 2015; Vuong C. K. et al., 2016). Below, we summarize how tissue-specific splicing regulatory RNA binding proteins influence splicing programs during neuronal development and how their dysregulation leads to neurological diseases.

**Table 1:** Neuronal alternative splicing regulatory RNA binding proteins (RBPs) and their target transcription and chromatin regulators.

RBP	RNA binding domain (RBD)	Number of RBDs	Binding elements in RNA	General mechanism of splicing	Target transcription and chromatin regulators
PTB proteins	RNA recognition motif (RRM)	4	CU-rich motifs	Promotes exon skipping	DPF2, PBX1
RBFOX proteins	RNA recognition motif (RRM)	1	(U)GCAUG	Promotes exon inclusion when binds downstream of alternative exon	–
				Promotes exon skipping when binds upstream of alternative exon	
NOVA proteins	(KH)-type RNA-binding domain	3	YCAY clusters	Promotes exon inclusion when binds downstream of alternative exon	LSD1
				Promotes exon skipping when binds upstream of alternative exon	
SRRM4	–	–	UGC containing motifs	Promotes exon inclusion when binds upstream of alternative exon	REST, MEF2C, MEF2D, TAF1, LSD1
Hu/ELAVL	RNA recognition motif (RRM)	3	U-rich and AU-rich motifs	Exon inclusion and exon skipping	–

“Y” represents a pyrimidine (C or U).

### Splicing regulation by PTB proteins

The polypyrimidine tract-binding protein (PTBP) family of splicing regulators, including PTBP1, PTBP2, and PTBP3, share structural and RNA-binding similarities but differ in cell type expression (Keppetipola et al., 2012; Spellman et al., 2007). PTBP1, also known as PTB, is widely expressed in most cell/tissue types except in neurons, muscle cells, and specific mature cells. The paralog PTBP2 (nPTB or brPTB) is found in neurons, myoblasts, and spermatocytes, while PTBP3 (ROD1) is expressed in hematopoietic and liver cells and does not affect neuronal splicing. Each PTB protein has four RNA recognition motif (RRM) domains that bind to extended CU-rich elements (Keppetipola et al., 2012). The two PTB proteins, PTBP1 and PTBP2, significantly influence post-transcriptional regulation during neuronal development (Boutz et al., 2007; Keppetipola et al., 2012; Nazim et al., 2024; Vuong C. K. et al., 2016; Vuong J. K. et al., 2016). By binding to CU-rich elements in pre-mRNAs, these proteins mainly repress a large number of exons but also stimulate splicing of some exons or cause retention of some introns (Hamid and Makeyev, 2017; Llorian et al., 2010; Yap et al., 2012; Yeom et al., 2021). Some exons maintain their repression through the switch from PTBP1 to PTBP2, while others, more sensitive to PTBP1, shift their splicing earlier when its expression level changes (Boutz et al., 2007; Li et al., 2014; Licatalosi et al., 2012; Linares et al., 2015; Zheng et al., 2012). Moreover, PTBP1 can dimerize and bridge RNA segments, causing looping out of exons or intronic segments to modulate exon splicing (Ye et al., 2023). The differential sensitivity of the two PTB paralogs may be due to exons requiring PTBP1 dimerization for repression, a property not seen in PTBP2.

PTBP1 is highly expressed in neural stem cells and progenitors but is sharply reduced upon mitotic exit by the induction of microRNA miR-124 (Makeyev et al., 2007). This reduction in PTBP1 level enhances miR-124 mediated repression of the REST complex (discussed below), a transcriptional suppressor of neuronal genes (Xue et al., 2013). Exons repressed by PTBP1 early in development affect functions such as axonogenesis, cell polarity, reduced apoptotic potential, and transcriptional programs of early neurons (Lin et al., 2020; Linares et al., 2015; Zhang M. et al., 2019). PTBP1 also represses exon 10 of the PTBP2 gene (Figure 1D), whose skipping leads to nonsense-mediated mRNA decay (NMD) of the PTBP2 transcript, preventing its expression in PTBP1-expressing cells (Boutz et al., 2007; Makeyev et al., 2007; Spellman et al., 2007). Reduced expression of PTBP1 during neuronal development derepresses exon 10 and allows PTBP2 expression, which is required for proper neuronal maturation. In contrast, PTBP2 exon 10 inclusion is promoted by the neural-specific SR-related protein SRRM4 in later stages of neuronal development, when PTBP1 expression is downregulated (Calarco et al., 2009). Additionally, PTBP1 represses the inclusion of many neural exons that are positively regulated by SRRM4, showing opposing regulation by these two RBPs during neuronal development (Raj et al., 2014).

Knockout of *Ptbp1* in mouse germline results in early embryonic lethality, implicating that many PTBP1 splicing targets are involved in maintaining pluripotency and inhibiting differentiation (Shibayama et al., 2009; Suckale et al., 2011). Pan-neuronal loss of Ptbp1 initially shows normal brain morphology but later shows progressive loss of ependymal cells in the lateral ventricles, leading to severe hydrocephaly and death by ~10 weeks of age (Shibasaki et al., 2013). One possibility is that the loss of PTBP1 may cause premature differentiation of radial glial cells into neurons, depleting the pool of radial glial cells necessary for generating ependymal cells (Spassky et al., 2005). In contrast, mice carrying germline null alleles or pan-neuronal conditional alleles of *Ptbp2* show perinatal lethality with respiratory failure and unresponsive to touch at birth (Li et al., 2014; Licatalosi et al., 2012). Depletion of PTBP2 in excitatory neurons of the dorsal telencephalon using an *Emx1-Cre* line showed similar brain morphology in *Emx1–Ptbp2^−/−^* brains compared to wild-type mice at birth, followed by cortical atrophy as early as P5, and extensive cell death and neuronal degeneration by P15 (Li et al., 2014). Moreover, *Ptbp2^−/−^* embryonic cortical neurons initially display similar plating efficiency and neurite outgrowth but show progressive cell death starting in the following weeks, possibly due to failed synapse formation or other maturation defects, contributing to perinatal lethality.

Interestingly, recent studies reported that depletion of PTBP1 or co-depletion of PTBP1 and PTBP2 were sufficient to induce the transdifferentiation of cells such as fibroblasts or astrocytes into fully mature neurons (Maimon et al., 2021; Qian et al., 2020; Xue et al., 2013; Zhou et al., 2020), although other groups have not replicated these findings (Chen et al., 2022; Hoang et al., 2022; Wang L. L. et al., 2021).

### Splicing regulation by RBFOX proteins

The highly conserved RBFOX family of RNA-binding proteins includes three paralogs: RBFOX1 (A2BP1), RBFOX2 (RBM9), and RBFOX3 (NeuN) with varying expression in different cell/tissue types (Conboy, 2017; Kuroyanagi, 2009). RBFOX1 and RBFOX2 are mainly expressed in neurons, skeletal muscle, and cardiac muscle, with RBFOX2 exhibiting a broader expression pattern across other tissues. In contrast, RBFOX3 is predominantly expressed in post-mitotic neurons. Upregulation of these splicing factors during neuronal development generally promotes the inclusion of many neuronal exons. RBFOX proteins contain a single high-affinity RRM domain that specifically recognizes and binds (U)GCAUG elements in pre-mRNA transcripts (Auweter et al., 2006; Jin et al., 2003; Lambert et al., 2014). Their splicing regulatory functions are context-dependent: binding to the downstream intron of an alternative exon typically promotes splicing, while binding to the upstream intron or within the alternative exon generally inhibits exon inclusion (Farshadyeganeh et al., 2023; Jangi et al., 2014; Lovci et al., 2013; Tang et al., 2009; Weyn-Vanhentenryck et al., 2014; Yeo et al., 2009; Zhang et al., 2008). Rbfox proteins are also part of a larger complex known as Large Assembly of Splicing Regulators (LASR) (Damianov et al., 2016; Ying et al., 2017). Within this complex, RBFOX can be indirectly recruited to RNA via interactions with other components like the hnRNP M and hnRNP H proteins, which partially explains why some of the RBFOX binding motifs identified in genome-wide assays do not contain a (U)GCAUG element (Peyda et al., 2024). This recruitment allows RBFOX to crosslink to RNA and function as a splicing regulator even in the absence of its typical (U)GCAUG binding motifs.

A large number of studies underscore the significant roles of RBFOX proteins in neuronal development and function from Drosophila to humans. In Drosophila, RBFOX-related genes were shown to regulate diverse developmental processes including germ cell differentiation and enhancing memory (Carreira-Rosario et al., 2016; Guven-Ozkan et al., 2016). Central Nervous System (CNS)-specific knockouts of *Rbfox1* or *Rbfox2* in mice exhibit distinct neurological phenotypes corresponding to their differential expression patterns in the cerebellum. *Rbfox1^−/−^* mice experience spontaneous seizures and heightened sensitivity to the neuroexcitatory agent kainic acid (Gehman et al., 2011). On the other hand, *Rbfox2^−/−^* mice have smaller cerebellums, abnormal Purkinje cell function, progressive motor difficulties, and often develop hydrocephalus early in life (Gehman et al., 2012). Exon-junction microarrays comparing the brains of *Rbfox1* and *Rbfox2* knockout mice to those of normal mice revealed significant splicing differences in alternative exons, many of which have adjacent (U)GCAUG motifs, suggesting they are direct targets of RBFOX proteins. Despite the complexity of correlating particular splicing changes to distinct phenotypes, some changes in ion channels and neurotransmitter genes in *Rbfox1* knockout mice were linked to the seizure phenotype. Notably, previous research indicated that splicing disruptions in genes such as *Gabrg2a* and *Grin1* have been associated with epilepsy in humans and altered seizure susceptibility in mice (Chapman et al., 1996; Gehman et al., 2011; Mulley et al., 2003; Zapata et al., 1997). Moreover, RBFOX1 expression is reduced in the post-mortem brains of individuals with autism, correlating with splicing irregularities in genes critical for synaptogenesis (Voineagu et al., 2011). Genome-wide mapping has shown that RBFOX1, RBFOX2, and RBFOX3 directly control the splicing of genes that are upregulated during brain development and whose dysregulation is linked to autism (Weyn-Vanhentenryck et al., 2014). Additionally, RBFOX1 regulates alternative splicing of an exon of the CaV1.2 voltage-gated calcium channel, affecting the channel’s electrophysiological properties in neurons (Tang et al., 2009). These observations collectively highlight the crucial role of RBFOX proteins in regulating splicing in neuronal development and function.

### Splicing regulation by NOVA proteins

The NOVA (neuro-oncologic ventral antigen) protein was first identified as an autoantigen in a neurological disease called paraneoplastic opsoclonus-myoclonus ataxia, characterized by motor and cognitive impairments (Buckanovich et al., 1993; Luque et al., 1991), and was the first RNA-binding protein described as a splicing regulator of neuron-specific exons (Jensen et al., 2000). NOVA1 and NOVA2, its two paralogs, each possess three K homology (KH)-type RNA-binding domains and bind to clusters of YCAY elements (Ule et al., 2006). The expression of NOVA proteins is upregulated during neuronal development. NOVA1 is mainly expressed in the ventral spinal cord and the hindbrain. In contrast, NOVA2 is predominantly expressed in the forebrain and dorsal spinal cord, with some overlapping expression in the midbrain and hindbrain regions (Yang et al., 1998). NOVA plays diverse roles in mRNA regulation, controlling alternative splicing and polyadenylation site selection to create brain-specific 3′ UTRs (Licatalosi et al., 2008; Ule et al., 2005). The binding of NOVA to an exonic YCAY cluster blocks U1 snRNP recruitment at the 5′ splice site (SS) and subsequently inhibits exon inclusion (Ule et al., 2006). Conversely, NOVA binding to a YCAY cluster in the downstream intron promotes spliceosome assembly and facilitates exon inclusion, whereas binding in the upstream intron generally inhibits exon splicing (Ule et al., 2006). These observations demonstrate a position-dependent regulation of splicing by NOVA (Ule et al., 2006). High-throughput sequencing data suggests that the regulatory network of NOVA encompasses a large number of alternative splicing events, including transcripts encoding synaptic proteins crucial for synaptic plasticity (Licatalosi et al., 2008; Zhang C. et al., 2010).

Genetic knockouts of *Nova1*, *Nova2*, or both have revealed their crucial roles in various aspects of brain development. *Nova1^−/−^* mice appear normal at birth but die within weeks of birth, exhibiting motor dysfunction, neuronal apoptosis, and action-induced tremors (Jensen et al., 2000). *Nova2^−/−^* mice shows mislocalization of neurons in different cortical layers and perturbed long-term potentiation of inhibitory postsynaptic current in hippocampal neurons (Yano et al., 2010). *Nova1/Nova2*-double knockout mice are paralyzed and die shortly after birth from respiratory failure (Ruggiu et al., 2009). The double knockout mice exhibit reduced acetylcholine receptor (AChR) clusters and a lack of alignment between AChR clusters and phrenic nerve terminals, which are not observed in single-knockout mice, suggesting that the NOVA proteins have redundant roles in regulating neuromuscular junction (NMJ) development and function. Altogether, these findings highlight the essential role of the NOVA proteins in the development and plasticity of the nervous system.

### Splicing regulation by SRRM4/nSR100

**Figure 2:**
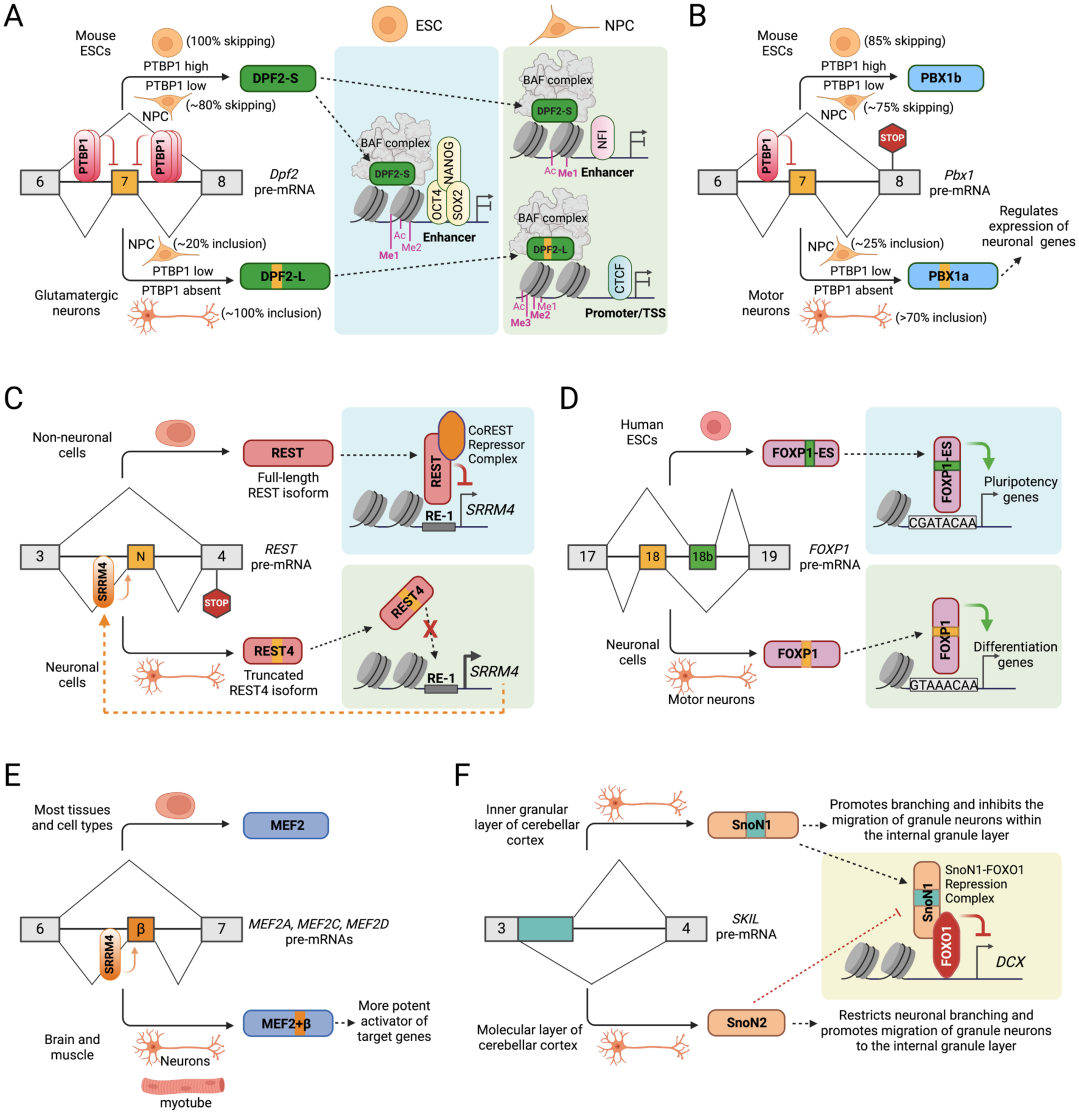
Alternative splicing of transcription factors in neuronal development. **(A)** PTBP1 regulated alternative splicing of *Dpf2* exon 7 alters the transcriptional and chromatin regulatory programs of stem cell maintenance and neuronal differentiation. **(B)** PTBP1 regulated alternative splicing of *Pbx1* exon 7 controls the expression of neuronal genes in motor neurons. **(C)** Cross-regulation between the neuronal alternative splicing activator SRRM4 and the transcription repressor REST controls the expression of neuronal genes. **(D)** Alternative splicing of mutually exclusive exons 18 and 18b in *FOXP1* gene controls the expression of pluripotency and differentiation genes in ESCs and motor neurons, respectively. **(E)** Brain- and muscle-specific inclusion of a microexon (β) in *MEF2A*, *MEF2C*, and *MEF2D* genes create a more potent activator of their target genes. **(F)** Alternative 5′ splice site selection in exon 3 of the *SKIL* gene to generate two SnoN isoforms that modulate neuronal branching and migration of granule neurons.

**Figure 3:**
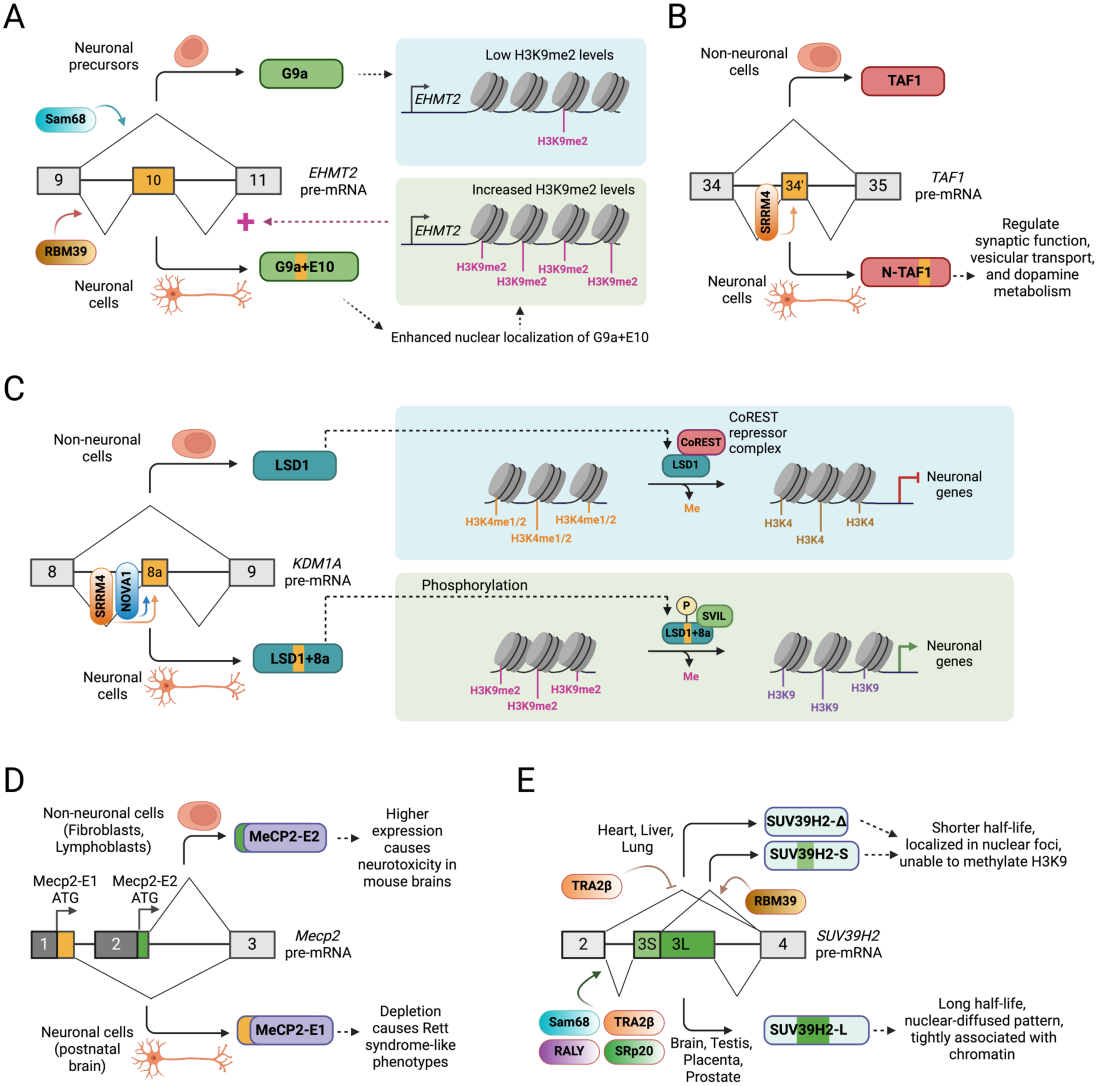
Alternative splicing of chromatin-modifying enzymes in neuronal development. **(A)** A positive feedback regulatory loop between histone methylation by histone methyltransferase G9a (EHMT2) and its alternative splicing regulation during neuronal development. **(B)** Alternative splicing of a 6 nucleotide microexon (exon 34′) in the *TAF1* creates a neuronal TAF1 isoform that regulates synaptic function, vesicular transport, and dopamine metabolism. **(C)** Alternative splicing of a 12 nucleotide microexon (8a) in histone demethylase LSD1 gene (*KDM1A*) allows its detachment from the CoREST repressor complex and the expression of neuronal genes. **(D)** Selection of alternative promoter exons create alternative MeCP2 isoforms with distinct N-terminus and biological functions. **(E)** Tissue-specific alternative splicing of exon 3 in *SUV39H2* gene generates multiple protein isoforms with distinct function and cellular localization.

The neural-specific SR-related protein SRRM4, also known as nSR100, is a vertebrate-specific splicing factor containing a Serine/Arginine-repeat region uniquely expressed in neurons across multiple brain regions and sensory organs (Calarco et al., 2009; Irimia et al., 2014; Quesnel-Vallières et al., 2015; Raj et al., 2014). Expression of SRRM4 increases during neuronal maturation (Irimia et al., 2014) and is essential for neurogenesis and neuronal differentiation, as demonstrated in mammalian cell cultures and zebrafish models (Calarco et al., 2009; Raj et al., 2014). SRRM4 is highly conserved among vertebrates but absent in invertebrates, suggesting that it likely emerged as an alternative strategy that evolved to support the enhanced regulatory complexities of the vertebrate nervous system (Torres-Méndez et al., 2022). SRRM4 promotes the inclusion of specific neuronal exons by recognizing UGC-containing motifs near the 3′ splice site and interacting with U2-RNP components to facilitate early spliceosome assembly (Raj et al., 2014). It regulates a network of brain-enriched alternative splicing events in genes crucial for neural functions, such as GTPase signaling, cytoskeletal organization, and synaptic membrane dynamics. Of particular interest is exon 10 of PTBP2 gene, which is repressed by its paralog PTBP1 in non-neuronal cells, causing the transcript to be targeted by NMD (Figure 1D). SRRM4 promotes the inclusion of *Ptbp2* exon 10, preventing its transcripts from undergoing NMD and promoting PTBP2 (nPTB) expression (Calarco et al., 2009). It also promotes the inclusion of a neural-specific exon in the transcription factor REST/NRSF, relieving its repressive effect and enhancing the expression of a subset of neural genes (Figure 2C) (Raj et al., 2011). Similarly, SRRM4 promotes the inclusion of neural microexons in several other transcription and chromatin regulators, including MEF2C, MEF2D, TAF1, and LSD1, which are discussed below (Figures 2E, 3B,C).

Loss of SRRM4 exhibits severe neuronal phenotypes in cultured cells, zebrafish, and mice. Depletion of SRRM4 in Neuro2a cells impairs neurite outgrowth, and affect neurosphere formation from differentiating ESCs or adult neural stem cells (Calarco et al., 2009). SRRM4 also promotes the inclusion of a microexon (exon L) in the protrudin pre-mRNA, resulting in a longer protrudin-L protein isoform that promotes neurite outgrowth (Ohnishi et al., 2017). In contrast, depletion of SRRM4 in Neuro2a cells suppresses the inclusion of exon L, resulting in the expression of a shorter protrudin-S isoform, which is less efficient in promoting neurite extension. One report showed that mutation in the *Srrm4* gene causes splicing defects and deafness in the sensory hair cells essential for hearing and balance in a Bronx Waltzer mouse model (Nakano et al., 2012). Knockdown of SRRM4 in zebrafish embryos shows severe neural degeneration and impaired axonal extension and branching (Calarco et al., 2009). In contrast, mice with SRRM4 haploinsufficiency exhibit severe neuronal phenotypes including altered neuronal excitability and synaptic transmission, and behavioral anomalies resembling autism spectrum disorder (Quesnel-Vallières et al., 2015, 2016). These observations highlight the essential functions of SRRM4 in the development of the nervous system.

### Splicing regulation by Hu/ELAVL

The Hu (also known as ELAVL) family of splicing regulators was first identified as autoantigens in a paraneoplastic neurological syndrome (Szabo et al., 1991). This family consists of four highly homologous members: HuA or HuR (ELAVL1), HuB (ELAVL2), HuC (ELAVL3), and HuD (ELAVL4) (Wei and Lai, 2022). While HuA is widely expressed in non-neural tissues, HuB, HuC, and HuD are predominantly found in neurons (Okano and Darnell, 1997) and are collectively known as neural ELAVLs (nELAVLs). Initially, Hu proteins have been shown to bind to U- and AU-rich elements in the 3′ UTR of mRNAs, enhancing their cytoplasmic stability and translation (Jain et al., 1997; Wang and Tanaka Hall, 2001). Further studies uncovered Hu proteins’ roles in alternative splicing of neuronal pre-mRNAs (Zhou et al., 2011; Zhu et al., 2006). Several studies have confirmed its roles in the alternative splicing and polyadenylation of genes related to neuronal function and diseases, such as *Bdnf* (Brain-derived neurotrophic factor) and *Nf1* (Neurofibromatosis type 1) (Allen et al., 2013; Zhu et al., 2008). Hu proteins interfere with U1 and U6 snRNP binding at the 5’ SS of an alternative exon (exon 23a) in the *Nf1* gene, whereas it causes decreased U2AF binding at the 3′ SS, thus influencing the alternative splicing outcome of the NF1 gene (Zhu et al., 2008). Moreover, Hu-mediated alternative polyadenylation generates differential 3′-UTRs that stabilize mRNAs in dendrites, facilitating local protein synthesis and contributing to synaptic plasticity (Allen et al., 2013; Bronicki and Jasmin, 2013; Zhou et al., 2011).

Regulation of RNA metabolism by Hu/ELAVL proteins is critically linked to neuronal differentiation and plasticity, as loss of nELAVLs in the brain results in various neurological abnormalities (Akamatsu et al., 2005; DeBoer et al., 2014; Ince-Dunn et al., 2012). HuC-null mice appear normal at birth and are fertile, but most adults exhibit impaired motor coordination, likely due to HuC being the sole nELAVL protein present in Purkinje cells (Ince-Dunn et al., 2012). These mice also experience non-convulsive electrographic seizures and spontaneous cortical hypersynchrony, possibly because of disrupted glutamate levels as nELAVLs bind to the 3′ UTRs of genes involved in glutamate synthesis. The prevalence of seizure phenotypes in other neuronal splicing regulator mutants, including HuC, suggests that many membrane and synaptic proteins are regulated through splicing. In contrast, HuD-null mice display motor and sensory neuron defects, particularly hind limb clasping, and a reduced number of cortical neurons despite an average count of neural stem cells (Akamatsu et al., 2005). Genome-wide profiling of nELAVL binding in HuC/HuD double-knockout brains has revealed hundreds of splicing changes regulated by nELAVL binding to specific intronic sites. Most of these splicing targets are associated with proteins that regulate microtubule dynamics at synapses and axons, suggesting crucial roles of Hu/ELAVLs in nervous system development and function (Ince-Dunn et al., 2012).

### Additional splicing regulatory proteins implicated in the nervous system

Several other RNA-binding proteins are also implicated in regulating alternative splicing in the nervous system. For instance, the RNA-binding protein RBM4 suppresses exon 9 of the *PTBP1* gene during neuronal differentiation of mesenchymal stem cells, resulting in a shorter PTBP1 isoform, PTBP1 (−E9), with significantly reduced splicing regulatory activity (Figure 1E), alleviating the repressive effect of PTBP1 on neuronal exons (Su et al., 2017). Interestingly, both RBM4 and PTBP1 prefer to bind CU-rich elements in pre-mRNA transcripts and antagonize each other’s function during differentiation. This functional antagonism is implicated in the alternative splicing regulation of pyruvate kinase M (PKM), where RBM4 antagonizes PTBP1 to promote a switch from the embryonic PKM2 isoform to the adult PKM1 isoform (Su et al., 2017). Additionally, RMB4 was shown to modulate alternative splicing of *Numb* exons 3 and 9 and promote neuronal differentiation and neurite outgrowth in mouse P19 cells (Tarn et al., 2016).

The KH-domain containing KHDRBS family of RNA-binding proteins, including SAM68 (KHDRBS1), SLM1 (KHDRBS2), and SLM2 (KHDRBS3), have been shown to control the splicing of neurexins, influencing synaptic functions (Vuong C. K. et al., 2016). The muscleblind-like 2 (MBNL2) splicing regulator, a member of the MBNL family of RNA-binding proteins, has been implicated in the neurological symptoms of myotonic dystrophy (Vuong C. K. et al., 2016). Another report showed that the RNA-binding proteins hnRNP H1 and H2 regulate the use of an alternative splice site of the telomere repeat-binding factor 2 (TRF2) pre-mRNA, encoding a shorter protein isoform (TRF2-S), a factor implicated in neuronal differentiation (Grammatikakis et al., 2016). On the other hand, mutations or dysfunction of TDP43 and FUS are associated with widespread splicing misregulation, which leads to neurodegenerative disorders such as amyotrophic lateral sclerosis (ALS) and frontotemporal lobar degeneration (FTLD) (Vuong C. K. et al., 2016). These studies highlight the diverse functional roles of different RNA-binding proteins in controlling the splicing regulatory programs in the nervous system.

## Alternative splicing of transcription factors in neuronal development

Among other tissues, the brain is particularly susceptible to splicing and transcriptional dysregulation, highlighting the necessity of studying neuron-specific splicing events in transcription regulators. One recent study developed a comprehensive transcriptome database for eight different cell types from the mouse cerebral cortex (neurons, astrocytes, microglia, oligodendrocyte precursors, newly formed oligodendrocytes, myelinating oligodendrocytes, endothelial cells, and pericytes) by RNA sequencing, identifying a large number of alternative splicing events that are cell type-specific, including genes encoding transcriptional regulators (Zhang et al., 2014). Another group created a manually curated database called “EpiFactors,” which includes expression data for various epigenetic regulators, their complexes, and targets (Medvedeva et al., 2015). By intersecting these two databases, Porter et al. (2018) identified 115 chromatin regulators exhibiting neuron-specific alternative splicing patterns. Additionally, comparing the EpiFactors dataset with a list of neuronally regulated microexons revealed 76 transcriptional regulators containing alternatively spliced microexons (Porter et al., 2018). The substantial number of transcriptional regulators undergoing neuron-specific alternative splicing events underscores their crucial role in the transcriptional regulation of neuronal development. Despite this, only a few studies have delved into the functional consequences of these alternative splicing switches. Below, we explore the role of alternative splicing in regulating transcription factor genes and its overall impact on neuronal development.

### Alternative splicing of the chromatin modifier DPF2

The mammalian chromatin-remodeling SWI/SNF complex (also known as BRG1/BRM-associated factor (BAF) complex) subunit DPF2 is a member of the BAF45 family of paralogous genes. The four BAF45 paralogs, including PH10 (BAF45a), DPF1 (BAF45b), DPF2 (BAF45d), and DPF3 (BAF45c), each contain two plant homeodomain (PHD) finger domains at the C-terminus, which facilitate the targeting of BAF complex to specific genomic loci bearing distinct histone marks and regulate gene transcription (Chestkov et al., 1996; Kadoch and Crabtree, 2015; Lessard et al., 2007). DPF2 is broadly expressed in different cell and tissue types and has been implicated in programmed cell death (apoptosis) in myeloid cells (Gabig et al., 1994), maintenance of pluripotency by interaction with pluripotency transcription factors in embryonic stem cells (Pardo et al., 2010; van den Berg et al., 2010), and in mesendodermal differentiation (Zhang W. et al., 2019). In a recent study, we reported that during neuronal differentiation, the DPF2 subunit switches from the canonical DPF2-Short (S) isoform to a longer DPF2-Long (L) isoform containing a new exon 7 (Figure 2A). In embryonic stem cells (ESCs), the splicing regulator PTBP1 suppresses *Dpf2* exon 7 to produce the DPF2-S isoform. Loss of PTBP1 during neuronal differentiation allows exon 7 inclusion, leading to the expression of the DPF2-L isoform (Nazim et al., 2024).

The two DPF2 isoforms differentially affect cellular phenotypes and transcriptional regulatory programs of ESCs, neural progenitor cells (NPCs), and glutamatergic neurons (GNs) (Nazim et al., 2024). Transcriptomic profiling in genome-edited mouse ESC lines that force expression of only DPF2-S or DPF2-L revealed that DPF2-S upregulates stem cell identity and pluripotency-associated genes such as *Lefty1*, *Lefty2*, *Myc*, *Zic2*, *Zic3*, *Wt1*, *Bmp4*, *Otx2*, *Lef1*, *Nodal*, and *Tcf15*, indicating its function in pluripotency maintenance. In contrast, DPF2-L upregulates neuron-specific genes in ESC-derived glutamatergic neurons, including *Vamp1*, *Syt2*, *Sncg*, *Nefh*, *Rph3a*, *Lynx1*, *Glra3*, *Hapln4*, and *Chrm2*, suggesting that DPF2-L modulates a subset of neuronal genes. Interestingly, forced expression of DPF2-L in ESCs exhibited flat-shaped colonies instead of the characteristic dome-shaped colonies, and a subpopulation of these cells showed reduced immunofluorescence of the stem cell pluripotency marker OCT4, indicating that DPF2-S is required for proper pluripotency maintenance in ES cells. In contrast, loss of DPF2-L in developing neurons that cannot switch to this isoform promotes the proliferation of an unidentified population of non-neuronal cells that do not stain for neuronal markers Map2 and GluR1, indicating that DPF2-L is required for proper glutamatergic differentiation (Nazim et al., 2024).

The two DPF2 isoforms exhibit overlapping but distinct binding preferences in chromatin (Nazim et al., 2024). DPF2-S preferentially targets chromatin regions bound by several stem cell pluripotency factors in ESCs, such as OCT4, SOX2, NANOG, ZIC2, and ZIC3. In NPCs, DPF2-S preferentially targets chromatin sites bound by NFI and several SOX proteins, while DPF2-L preferentially targets sites bound by CTCF and BORIS (CTCFL), suggesting that alternative DPF2 isoforms differentially target regulatory regions in NPCs. Moreover, the DPF2-S and -L preferential binding sites are marked by distinct chromatin modifications (Nazim et al., 2024). DPF2-S binds to chromatin sites with enhancer-specific modifications, including H3K4me1, H3K4me2, and H3K27ac, while DPF2-L binds to sites enriched for promoter modifications, including H3K4me3, H3K9ac, H3K4me2, and H3K27ac. These findings show that the timely alternative splicing switch of the highly conserved *Dpf2* exon 7 is critical in regulating BAF function and epigenetic programs during neuronal development.

### Alternative splicing of the transcription factor PBX1

The pre-B-cell leukemia homeobox transcription factor 1 (PBX1) belongs to the PBX1-4 family, which regulates diverse developmental programs, including cell proliferation and differentiation, malignant cell transformation, and apoptosis (Bourette et al., 2007; Dedera et al., 1993; Smith et al., 1997; Sykes and Kamps, 2004). PBX1 forms heterodimers with Hox homeodomain proteins to bind DNA/chromatin to promote gene transcription (Charboneau et al., 2006; LaRonde-LeBlanc and Wolberger, 2003; Piper et al., 1999). A conserved exon 7 in *Pbx1* is alternatively spliced during neuronal development (Linares et al., 2015). In early embryonic tissues, high expression of splicing regulatory protein PTBP1 represses exon 7 to generate the PBX1b isoform, where the translational reading frame is shifted to introduce a premature termination codon (PTC) in exon 8 (Figure 2B). The PTC does not result in Nonsense-mediated mRNA decay (NMD) but instead generates the shorter protein isoform, which lacks 83 amino acids at the C-terminus but retains the DNA binding homeodomain. In neural tissues, PTBP1 expression is downregulated, which allows the inclusion of exon 7 to generate the PBX1a isoform. PBX1 is thus a target of the larger PTBP1 regulatory program in neuronal development (Linares et al., 2015).

Interestingly, deletion of intronic regions to eliminate PTBP1 binding sites upstream to exon 7 upregulates PBX1a expression in ESCs. Differentiation of these mutant ESCs into motor neuron lineage induces a subset of neuronal genes involved in axonogenesis, regulation of transcription, pattern specification, cell fate commitment, cell adhesion, cell motion, and heart development as early as 2 days in culture, indicating that early expression of PBX1a activates the neuronal transcriptional program (Linares et al., 2015). Roughly a quarter of the PBX1a-induced genes also exhibited nearby PBX1 binding. Interestingly, several transcription factors with neuronal functions, including the homeobox C5 transcription factor (Hoxc5), were among the upregulated genes. The upregulation of Hoxc5 in motor neurons is potentially regulated by increased binding of PBX1 and its cofactor Meis1 at the Hoxc5 locus. These findings suggest that the alternative splicing of *Pbx1* exon 7 is critical in determining neuronal fate during differentiation.

### Alternative splicing of the transcription factor REST/NRSF

The Neuron-Restrictive Silencer Factor (NRSF), commonly referred to as RE-1 Silencing Transcription factor (REST), was first identified in non-neuronal tissues where it represses neuronal genes (Chong et al., 1995). REST binds to RE-1 elements located in the promoter regions of specific neuronal genes and recruits a co-repressor complex, facilitating suppression of neuronal genes (Bruce et al., 2004; Chen et al., 1998; Schoenherr and Anderson, 1995). The splicing regulatory protein SRRM4 (nSR100) promotes the inclusion of a 16-nucleotide microexon between exons 3 and 4 in the REST gene, producing the neuron-specific “REST4” isoform (Figure 2C) (Palm et al., 1999; Raj et al., 2011). In non-neuronal cells, skipping of this microexon ensures full-length REST protein expression. However, in neuronal cells, the inclusion of this microexon changes the reading frame and generates a premature termination codon in exon 4, ultimately resulting in a truncated REST4 protein isoform lacking four zinc finger domains and a C-terminal repressor domain, which are required for DNA binding and gene repressive activities, respectively. The shorter REST4 protein may also act in a dominant-negative manner by sequestering full-length REST into nonfunctional hetero-oligomers, relieving the suppressive effect of REST on neuronal genes (Shimojo et al., 1999).

In non-neuronal cells, REST directly represses nSR100 expression, creating a regulatory loop that maintains the downregulation of neuronal genes. In neurons, expression of nSR100 is upregulated as neuronal differentiation progresses, leading to the microexon inclusion that produces the REST4 isoform with significantly reduced repressive activity and, therefore, activating the expression of REST targets in neural cells (Raj et al., 2011). Although overall REST expression is decreased in neurons, nSR100-mediated alternative splicing ensures complete loss of REST function and expression of neuronal genes. Intriguingly, the loss of nSR100 expression in the developing mouse brain disrupts neurogenesis, consistent with the crucial role of nSR100 in inhibiting REST activity (Raj et al., 2011). These findings emphasize the antagonistic molecular relationship between the transcriptional repressor REST and the neuronal splicing activator nSR100, which is crucial for maintaining the identity of neuronal and non-neuronal cells.

### Alternative splicing of the transcription factor FOXP1

Forkhead Box P1 (FOXP1) is one of four members of the FOXP subfamily of transcription factors that regulate numerous genes involved in cell proliferation, differentiation, and development (Wijchers et al., 2006). The forkhead domain of FOXP proteins is known to bind a canonical consensus motif GTAAACA on its target genes as either a monomer or homo- and/or heterodimers. Previous studies have shown that knockout of *Foxp1* in mice disrupts the establishment of specific cell types and results in early embryonic lethality (Dasen et al., 2008; Wang et al., 2004; Zhang Y. et al., 2010). In human pluripotent ESCs, a highly conserved exon 18b in the *FOXP1* transcript becomes included instead of exon 18, whereas exon 18 is included in other differentiated cell lines (Figure 2D). Similarly, mouse *Foxp1* exon 16 but not exon 16b (orthologous exons 18 and 18b in humans) is included during ESC differentiation into embryoid bodies or motor neurons (Gabut et al., 2011). The inclusion of exon 18b (FOXP1-ES) in ESC maintains the reading frame but alters critical amino acid residues within the forkhead domain. Interestingly, protein-binding microarray analysis showed that FOXP1 and FOXP1-ES forkhead domains prefer distinct DNA-binding motifs. While FOXP1 predominantly recognizes and binds the canonical binding motif GTAAACAA, FOXP1-ES prefers CGATACAA or closely related sequences (Gabut et al., 2011). These findings suggest that the specific inclusion of exon 18b in human ESCs modifies the DNA-binding specificity of FOXP1.

Alternatively spliced FOXP1 isoforms regulate distinct programs of gene expression in human ESCs. In undifferentiated human ESCs, the two FOXP1 isoforms regulate distinct and overlapping sets of target genes, although FOXP1-ES regulates a larger set of genes than FOXP1. The altered DNA-binding specificity switches the transcriptional output of FOXP1-ES such that the pluripotency genes *OCT4*, *NANOG*, *GDF3*, *NR5A2*, and *TDGF1* are stimulated while genes involved in cell-lineage specification and differentiation are repressed. Moreover, induced expression of the FOXP1-ES isoform inhibits neural cell differentiation and promotes ESC self-renewal and pluripotency maintenance. In contrast, the mouse Foxp1-ES is required for efficient reprogramming of mouse embryonic fibroblasts (MEFs) into iPSCs. Thus, alternative splicing of an evolutionarily conserved exon reconfigures transcriptional regulatory programs required for ESC self-renewal, pluripotency maintenance, and neuronal differentiation.

### Alternative splicing of the transcription factor MEF2

Myocyte Enhancer-binding Factor 2 (MEF2), also known as MADS box transcription enhancer factor 2, is a family of four paralogous transcription factors, including MEF2A, MEF2B, MEF2C, and MEF2D, which are involved in the development of both the muscle and nervous system. Notably, MEF2 factors have previously been shown to regulate genes associated with synapse development (Flavell et al., 2008; Flavell et al., 2006). Previous studies have also identified that the MEF2C gene exhibits alternative pre-mRNA splicing at multiple sites, resulting in various isoforms, including some that are brain-specific (Janson et al., 2001; Leifer et al., 1993). Interestingly, three MEF2 family members, MEF2A, MEF2C, and MEF2D, have a highly conserved 24-nucleotide exon encoding a short domain designated as *β* (Figure 2E), that is only expressed in striated muscle and neurons (Leifer et al., 1993; Zhu et al., 2005). Multiple reports showed that SRRM4 directly regulates the inclusion of this microexon in *MEF2C* and *MEF2D* by binding to UGC motifs adjacent to the polypyrimidine tract upstream of the alternative exon (Raj et al., 2014; Torres-Méndez et al., 2022). Reporter assays show that the inclusion of the β domain, which is adjacent to the MEF2 transactivating domains, creates a more potent activator of MEF2 target genes (Zhu et al., 2005). The authors showed that the observed activity is not attributable to *cis* effects on MEF2 DNA binding or dimerization, nor does it involve interactions with established transcription factors or coactivators, but instead generates an acidic activation domain selectively in muscle and neurons.

### Alternative splicing of the transcription factor SKIL/SnoN

The transcription factor SKI-like proto-oncogene (SKIL), also known as SnoN, plays a vital role in axon morphogenesis in the cerebellar cortex (Ikeuchi et al., 2009; Stegmüller et al., 2006). The SnoN gene undergoes alternative splicing, where activation of the canonical 5’ SS produces the full-length SnoN1 isoform, while activation of an alternative 5’ SS within exon 3 results in a 46 amino acid deletion, generating the shorter SnoN2 isoform (Figure 2F) (Pelzer et al., 1996). Both SnoN isoforms function in neurons, but their roles are confined to specific cerebellar layers (Huynh et al., 2011). SnoN1 is predominantly found in the inner granular layer, while SnoN2 is primarily expressed in the molecular layer.

Interestingly, SnoN1 and SnoN2 exhibit opposing functional roles in coordinating neuronal branching and positioning (Huynh et al., 2011). Knockdown of SnoN1 results in the suppression of neural branching but promotes the migration of granule neurons in the cerebellar cortex, while knockdown of SnoN2 produces the opposite effect. Intriguingly, SnoN1, but not SnoN2, can form a complex with the transcription factor FOXO1 and repress the expression of doublecortin (DCX) in cerebellar granule neurons (Figure 2F), thereby controlling neuronal branching and positioning (Huynh et al., 2011). These observations highlight an isoform-specific SnoN1-FOXO1 complex that orchestrates the transcriptional regulation of neuronal branching and positioning in the brain.

## Alternative splicing of chromatin-modifying enzymes in neuronal development

Chromatin-modifying enzymes are pivotal in maintaining the chromatin architecture, influencing the accessibility of the transcriptional machinery, and thereby regulating gene expression. A significant number of these enzymes (histone Readers, Writers, Erasers) undergo neuron-specific alternative splicing, producing isoforms essential for the epigenetic regulatory programs involved in neurodevelopment (Porter et al., 2018). The resulting isoforms from these splicing events play crucial roles in shaping the chromatin landscape and transcriptional regulatory programs during neuronal development. Below, we delve into how these neuron-specific alternative splicing events impact the regulation of chromatin and transcriptional processes in neuronal development.

### Alternative splicing of histone methyltransferase EHMT2/G9a

The histone methyltransferase (HMTase) EHMT2, also known as G9a, belongs to a family of six members, including GLP (EHMT1), SETDB1, SETDB2, SUV39H1, and SUV39H2. These HMTases control the mono-, di-, or tri-methylation of histone H3 at lysine 9 (H3K9me1/2/3) (Fritsch et al., 2010), histone marks generally associated with transcriptional repression (Kouzarides, 2007). G9a plays a critical role in the differentiation of various cell and tissue types, including tenocyte growth and differentiation (Wada et al., 2015), skeletal muscle differentiation (Ling et al., 2012), differentiation of monocyte and T helper cells (Lehnertz et al., 2010; Wierda et al., 2015), cardiac development (Inagawa et al., 2013), and maturation of gametes (Tachibana et al., 2002). G9a has also been implicated as a critical regulator in pluripotent stem cells and the nervous system. G9a promotes specific gene silencing by local heterochromatinization through a pronounced increase in histone H3K9 methylation (H3K9me1/2), which causes irreversible epigenetic inactivation of pluripotency transcription factors Oct-3/4 and prevents reprogramming of ESCs during differentiation (Epsztejn-Litman et al., 2008; Feldman et al., 2006). In the nervous system, G9a is crucial for controlling cognition and adaptive behavior in mice, indicating that G9a-mediated histone H3K9 di-methylation is essential for regulating brain function by maintaining transcriptional homeostasis in adult neurons (Schaefer et al., 2009). In Drosophila, G9a regulates peripheral dendrite growth, classical learning, and expression of memory-related genes (Kramer et al., 2011). G9a has also been shown to affect the specification of different neuronal subtypes in the striatum (Maze et al., 2014) and the regulation of ethanol-induced neurodegeneration in neonatal mice brains (Subbanna et al., 2013).

Accumulating evidence has shed light on the role of alternative splicing of G9a in neuronal development and function. The existence of two alternatively spliced transcripts of G9a with the presence or absence of exon 10 was first described in 2001 (Brown et al., 2001). More recent reports show that G9a exon 10 is alternatively spliced in a tissue-specific and developmental-stage-specific manner (Fiszbein et al., 2016; Mauger et al., 2015). SiRNA-mediated depletion experiments suggest that Sam68 represses, but RBM39 promotes G9a exon 10 inclusion in HeLa, MCF7, and SKOV3-ip cells (Mauger et al., 2015). The inclusion of G9a exon 10 generates a longer protein isoform without altering the organization of G9A protein domains (Figure 3A). The methyltransferase activity of G9a is required for proper neuronal differentiation of N2a cells in culture, and exon 10 inclusion increases during neuronal differentiation (Fiszbein et al., 2016; Fiszbein and Kornblihtt, 2016). Exon 10 inclusion does not affect the intrinsic catalytic activity of G9a but results in increased global levels of H3K9me2. This is in part due to the higher nuclear localization of G9a containing exon 10, although the mechanism of its nuclear localization is unclear. Interestingly, G9a methylates its own intragenic histone marks, leading to a more compact chromatin structure, which subsequently promotes the inclusion of exon 10 (Figure 3A). The data imply a positive feedback loop highlighting the crucial roles of alternatively spliced isoforms of G9a in cellular commitment to differentiation.

### Alternative splicing of histone acetyltransferase TAF1

The histone acetyltransferase TAF1 is a TFIID transcription initiation complex component that recruits RNA Polymerase II to transcription start sites (TSS) (Jacobson et al., 2000; Mizzen et al., 1996). A six-nucleotide microexon (34′) close to the two bromodomains of TAF1 near the C-terminus is alternatively spliced during neuronal maturation to create the neuronal TAF1 isoform, also known as N-TAF1 (Figure 3B) (Ito et al., 2016; Jambaldorj et al., 2012). A recent report showed that the *TAF1* neural microexon inclusion is directly regulated by SRRM4 through the recognition of UGC elements upstream of the regulated microexon (Capponi et al., 2020). Interestingly, depletion of N-TAF1 in neuroblastoma cells downregulates genes involved with synaptic function, vesicular transport, and dopamine metabolism, suggesting its essential roles in the nervous system (Herzfeld et al., 2013). The N-TAF1 isoform has been implicated in X-linked Dystonia-Parkinsonism (XDP), an adult-onset neurodegenerative disorder presenting features of dystonia and parkinsonism. XDP is caused by a ~ 2.6 kb SINE-VNTR-Alu (SVA)-type retrotransposon insertion into intron 32 of the *TAF1* gene (Domingo et al., 2015; Makino et al., 2007; Nolte et al., 2003). XDP patient-derived neural cells show significantly reduced expression of the N-TAF1 protein, suggesting that the SVA retrotransposon may disrupt the expression of N-TAF1 in neurons (Makino et al., 2007). Recent studies also identified that the SVA insertion into intron 32 of the *TAF1* gene generates a partially intron-retained (IR) aberrant RNA transcript that reduces exon usage in proximity to the SVA and overall TAF1 expression in patient-derived neural cells (Aneichyk et al., 2018). However, the molecular mechanisms leading to partial intron 32 retention, whether the SVA insertion has additional effects on RNA metabolism, and the ultimate fate of the mutant *TAF1* mRNA remain unclear. Moreover, multiple point mutations and duplications in the *TAF1* gene were implicated in X-linked intellectual disability in males, presenting various neurological features, although the molecular mechanism of pathogenesis remains poorly understood. Altogether, the data suggests a vital role of the alternatively spliced N-TAF1 isoform in neurons and warrants further functional studies in both *in vitro* and *in vivo* settings to address its specific function in normal physiology and genetic diseases.

### Alternative splicing of histone demethylase KDM1A/LSD1

Lysine (K)-Specific Demethylase 1A (KDM1A), commonly known as LSD1, is a histone-modifying enzyme that demethylates mono- and di-methylated lysine 4 residues on histone H3 (H3K4me1 and H3K4me2), leading to repression of target genes (Shi et al., 2004). The canonical KDM1A was found to be an essential component of the CoREST repressor complex that represses neuronal genes in non-neuronal cells (discussed above) (Ballas et al., 2001; Shi et al., 2004).

The LSD1 gene has two alternatively spliced exons, namely, E2a (60 bp long) and E8a (12 bp long), whose inclusion does not alter the reading frame of the LSD1 protein. In neurons, the 12 nucleotide E8a microexon is included to produce a neuronal isoform (Figure 3C) (Zibetti et al., 2010). The newly encoded four amino acids by E8a (with sequence Asp-Thr-Val-Lys) immediately precede the CoREST-binding domain of LSD1. Multiple lines of evidence suggest that the expression of the neuronal LSD1 isoform (LSD1 + 8a) is upregulated during neuronal maturation, which plays essential roles in synaptogenesis and neurite morphogenesis and ensures proper transcriptional response to neuronal depolarization (Laurent et al., 2015; Toffolo et al., 2014; Wang et al., 2015; Zibetti et al., 2010). It was also shown that in neuronal cells, the splicing regulatory proteins NOVA1 and SRRM4 binds to LSD1 pre-mRNA and promote the inclusion of exon 8a (Rusconi et al., 2015). Knockdown of LSD1 + 8a isoform in mouse cortical neurons inhibits, whereas its overexpression promotes neurite morphogenesis (Toffolo et al., 2014; Zibetti et al., 2010). In contrast, LSD1 exon 8a limited-knockout mice display reduced neuronal excitability and are less susceptible to seizures (Rusconi et al., 2015). One report showed that the LSD1 + 8a interacts with the nuclear factor supervillin (SVIL) and demethylates the repressive H3K9me2 mark but loses its intrinsic capability to demethylate H3K4me2 and, therefore, function as an activator of its target genes (Figure 3C) (Laurent et al., 2015). Interestingly, phosphorylation of the threonine residue at position 369 encoded by the neuronal exon causes a conformational change that leads to its detachment from the CoREST complex (Toffolo et al., 2014). Another report suggests that the LSD1 + 8a acquires a unique substrate specificity to demethylate H4K20me1/2, a histone mark associated with transcriptionally repressed chromatin regions, and regulates the expression of genes related to learning and memory formation (Wang et al., 2015). Collectively, these findings highlight the critical functional roles of the neuronal splice variant of LSD1 in the nervous system.

### Alternative splicing of methyl DNA reader MeCP2

The methyl-CpG binding protein 2 (MeCP2) is highly expressed in neurons and functions as an epigenetic silencer by binding to methylated CpG sites and interacting with the corepressor SIN3A (Amir et al., 1999; Jones et al., 1998; Nan et al., 1998). Loss-of-function mutations in the *MECP2* gene typically result in a pediatric neurodevelopmental disorder called Rett syndrome, which affects young females exhibiting clinical features such as intellectual impairment, reduced language and motor skills, and hand stereotypies (Amir et al., 1999; Pohodich and Zoghbi, 2015). Maintenance of appropriate levels of MeCP2 is crucial for normal brain function.

The splicing of alternative first exons in the *MECP2* gene generates two distinct isoforms: one that encodes a 21 amino acid peptide (MeCP2-E1) and another encoding a nine amino acid peptide (MeCP2-E2) at the N-terminus of the protein (Figure 3D) (Kriaucionis and Bird, 2004; Mnatzakanian et al., 2004). The MeCP2-E1 isoform is expressed at higher levels than the MeCP2-E2 isoform in postnatal brains (Dragich et al., 2007; Zachariah et al., 2012). The alternative N-terminal peptides are positioned close to the Methyl-Cytosine Binding Domain (MBD), potentially affecting its ability to bind to methyl-CpG sites. In parallel work, two groups showed that knockout of *Mecp2* in mice results in Rett syndrome-like phenotypes (Chen et al., 2001; Guy et al., 2001). Interestingly, the deletion of MeCP2-E1 in mice recapitulated the neurological features associated with Rett syndrome (Yasui et al., 2014), but the deletion of MeCP2-E2 did not show these neurological features (Itoh et al., 2012). These data suggest that the haploinsufficiency of the MeCP2-E1 variant is specifically associated with Rett syndrome. In contrast, higher levels of the MeCP2-E2 isoform, but not the MeCP2-E1 isoform, show neurotoxicity in mouse brains (Dastidar et al., 2012). Interestingly, MeCP2-E2 can directly interact with the transcription factor FoxG1, which inhibits the MeCP2-E2 mediated neurotoxicity. These observations suggest that the two alternatively spliced MeCP2 isoforms play different functional roles in the nervous system.

### Alternative splicing of histone methyltransferase SUV39H2

SUV39H2 and its paralog SUV39H1 are histone methyltransferases that catalyze the H3K9me3 mark. SUV39H2 was initially described as an early embryonic (embryonic stem cells, embryoid bodies, and early mouse embryos) and adult testis-specific protein (O’Carroll et al., 2000). However, the study of *Suv39h1* knockout and *Suv39h1/Suv39h2* double-knockout mice indicates that it may have functions in other tissues (Peters et al., 2001). SUV39H2 is a ubiquitously expressed protein, but in adult tissues, the expression is enriched in the cerebellum and testis (Weirich et al., 2021). SUV39H2 promotes the maintenance of trophoblast stem cells, restrains trophoblast cell differentiation, and contributes to the epigenetic landscape of placental development (Wang L. et al., 2021). Another study has shown that the knockdown of SUV39H2 inhibits stemness and cell proliferation of glioma cells and promotes their chemosensitivity (Wang et al., 2019). Recent studies have also shown that SUV39H1 and SUV39H2 control the differentiation of NPCs in the adult hippocampus (Guerra et al., 2022). Another study identified a loss-of-function variant of SUV39H2 in autism-spectrum disorder that causes altered H3K9 trimethylation and dysregulation of protocadherin *β*-cluster (Pcdhb cluster) genes in the developing brain (Balan et al., 2021). These observations delineate a critical role of SUV39H2 in the nervous system.

A study by Mauger et al. (2015) showed a broad expression pattern of SUV39H2 in different human tissues, including the brain. The authors showed that SUV39H2 exon 3 is alternatively spliced in a tissue-specific manner, where exon 3 can be skipped (SUV39H2-*Δ*), partially included (SUV39H2-S) using a cryptic 5′ splice site, or fully included (SUV39H2-L) (Figure 3E). Multiple RNA-binding proteins, including Sam68, RALY, TRA2β, SRp20, RBM9, and RBM39, modulate the alternative splicing of exon 3. Like the G9a protein, SUV39H2 protein also contains an evolutionarily conserved SET domain required for their HMTase activities. Total or partial skipping of *SUV39H2* exon 3 causes a large deletion in the SET domain (in SUV39H2-S and SUV39H2-Δ isoforms) and in the chromodomain (in SUV39H2-Δ isoform) that binds methylated H3K9. The shorter SUV39H2 isoforms (SUV39H2-S and SUV39H2-Δ) show a shorter half-life in protein stability assays, suggesting that exon 3 inclusion determines SUV39H2 protein stability. The inclusion of exon 3 also regulates SUV39H2 sub-nuclear localization, where the full-length SUV39H2-L shows a nuclear-diffused pattern, but SUV39H2-S and SUV39H2-Δ isoforms are concentrated in the nuclear foci (Mauger et al., 2015). Biochemical fractionation of HeLa cells showed that the longer SUV39H2-L isoform does not co-fractionate with the shorter isoforms. SUV39H2-L is codistributed with H3 and heterochromatin protein 1α (HP1α), suggesting it is more tightly associated with chromatin than the shorter isoforms. The differential distribution of alternative SUV39H2 isoforms in the chromatin may indicate that they are involved in different complexes (Mauger et al., 2015). *In vitro* methylation assay indicates that the SUV39H2-S and SUV39H2-Δ isoforms, lacking a full-length SET domain, are unable to methylate H3K9, suggesting that the skipping of exon 3 affects its H3K9 methyltransferase activity. Moreover, alternative splicing of *SUV39H2* exon 3 was also shown to regulate various target genes. Transcriptomic profiling of HeLa cells expressing exogenous SUV39H2-L and -S isoforms showed that a subset of target genes was differentially regulated by the two isoforms, suggesting that the ratio between the alternatively spliced SUV39H2 isoforms is crucial for the normal regulation of their target genes. Further ChIP assays revealed that the promoter regions of some of the target genes were occupied by SUV39H2-L, indicating that the full-length isoform acts directly on the promoters of its target genes. Altogether, the data suggests that the alternative splicing of SUV39H2 generates protein isoforms with different tissue-specific functions.

## Perspectives

Alternative splicing of pre-mRNA transcripts is highly prevalent in vertebrates. The brain, in particular, exhibits the most intricate patterns of alternative splicing, producing a wide array of protein isoforms not typically found in other tissues. Recent high-throughput transcriptomic profiling has identified numerous neuronal alternatively spliced exons regulated by specialized neuron-specific splicing regulatory proteins/programs, resulting in isoforms with distinct functions. Among the many hundreds of RNA-binding proteins, only a few have been implicated in controlling neuronal splicing programs so far. It is likely that other RNA-binding proteins, yet to be analyzed in detail, also contribute to the neuronal splicing programs, adding further layers of complexity to gene regulation in the brain. While the functions of some alternatively spliced variants of transcription and chromatin regulators have been studied in greater detail, many alternative splicing events still need to be examined. As highlighted in this review, understanding the functional consequences of these events is crucial for fully grasping the various aspects of neuronal development and function, as well as comprehending the pathomechanisms of related neurodevelopmental disorders.

Emerging genetic tools and advanced next-generation sequencing technologies will aid future researchers in providing a more detailed understanding of the dynamic role of splicing programs in determining cell fate and differentiation of stem/progenitor cells into various neuronal lineages and the development of neural circuits. The study of splicing factors in knockout models is complicated due to their highly pleiotropic effects, as these modulations are often lethal or result in developmental defects that mask functions that would appear later in development. To circumvent this, prior studies have used Cre recombinase-expressing conditional knockout mouse lines. This strategy allows the depletion of specific genes in specific tissue or cell types and at specific time points, which is particularly advantageous for studying the function of regulatory proteins in different tissues and developmental stages. However, knocking out specific regulators can affect many target genes involved in common biological pathways, making it difficult to link specific phenotypes with specific splicing events or variants. One approach to circumvent this limitation is to modulate genes by techniques such as CRISPR/Cas9-mediated gene-editing so that cells can generate one particular splice variant and not the other. This methodology has been used to study the function of specific splice variants in genes such as *Dpf2* and *Mecp2* (discussed earlier), where researchers modulated target genes to allow the expression of specific isoforms of these proteins.

Recent advancements in single-cell/nuclei RNA sequencing (sc/snRNA-seq) technologies provided unparalleled advantages for examining the individual cell-level transcriptome, revealing cellular heterogeneity that bulk RNA-seq often obscures. This is especially valuable in complex tissues like the brain, where diverse cell types and states coexist. Additionally, sc/snRNA-seq can trace cell lineage and differentiation pathways, offering unique insights into the development of various cell types, which is crucial for understanding cellular and tissue development. When combined with spatial transcriptomics or time-course studies, scRNA-seq can demonstrate how gene expression varies across tissue regions or changes over time, offering a dynamic perspective on cellular processes. However, analysis of isoform-specific expression driven by alternative splicing is particularly challenging due to factors such as uneven or low capturing of the transcript coverage from single cells, variability in the number of RNA molecules in cells, number of cells sequenced, low cDNA conversion efficiency, and sequencing errors and artifacts, which often result in low coverage and high technical noise. However, recent advances in single-cell long-read sequencing enabled researchers to distinguish isolated and coordinated alternative splicing events and assign the events to the cell of origin. The utilization of genetically engineered fluorescent proteins and cell-surface markers, combined with fluorescence-activated cell sorting (FACS), has made it possible to isolate different cell types of the neuronal lineage, including neural progenitor cells and specific neuronal subtypes. Another method that can be used to capture cell-type specific splicing signatures is the utilization of Ribo-Tag/TRAP, where a tag is added to a protein of the large ribosomal subunit. This method is particularly useful for analyzing ribosome-bound/translating mRNAs in particular cells expressing the tagged ribosomal protein. Improved single-cell fluorescence *in situ* hybridization (FISH) and immunofluorescence (IF) methods also have the potential to uncover topological alterations in alternative splicing within the brain network. Additionally, spatial transcriptomic techniques, such as multiplexed error-robust fluorescence *in situ* hybridization (MERFISH), could be highly effective for characterizing the expression and spatial distribution of alternative spliced transcripts in a high-throughput manner. These cutting-edge molecular genetic tools will enable future researchers to explore gene regulation in the nervous system with unprecedented precision and depth, providing new insights into the complexities of neural gene expression and function.
